# Evaluation of the Productivity and Potential Utilization of *Artemisia dubia* Plant Biomass for Energy Conversion

**DOI:** 10.3390/plants13081158

**Published:** 2024-04-22

**Authors:** Algirdas Jasinskas, Gintaras Šiaudinis, Danutė Karčauskienė, Renata-Marks Bielska, Marek Marks, Stanislaw Bielski, Ramūnas Mieldažys, Kęstutis Romaneckas, Egidijus Šarauskis

**Affiliations:** 1 Department of Agricultural Engineering and Safety, Faculty of Engineering, Vytautas Magnus University, Agriculture Academy, Studentu Str. 15A, Akademija, LT-53362 Kaunas, Lithuania; ramunas.mieldazys@vdu.lt (R.M.); egidijus.sarauskis@vdu.lt (E.Š.); 2 Vėžaičiai Branch, Lithuanian Research Centre for Agriculture and Forestry Vėžaičiai, LT-96216 Klaipėda, Lithuania; gintaras.siaudinis@lammc.lt (G.Š.); danute.karcauskiene@lammc.lt (D.K.); 3 Department of Economic Policy, Faculty of Economics, University of Warmia and Mazury in Olsztyn, M. Oczapowskiego 8, 10-719 Olsztyn, Poland; renata.bielska@uwm.edu.pl; 4 Department of Agroecosystems and Horticulture, Faculty of Agriculture and Forestry, University of Warmia and Mazury in Olsztyn, M. Oczapowskiego 8, 10-719 Olsztyn, Poland; marek.marks@uwm.edu.pl; 5 Department of Agrotechnology and Agribusiness, Faculty of Agriculture and Forestry, University of Warmia and Mazury in Olsztyn, M. Oczapowskiego 8, 10-719 Olsztyn, Poland; stanislaw.bielski@uwm.edu.pl; 6 Department of Agroecosystems and Soil Science, Faculty of Agronomy, Vytautas Magnus University, Agriculture Academy, Studentu Str. 11, Akademija, LT-53361 Kaunas, Lithuania; kestutis.romaneckas@vdu.lt

**Keywords:** *A. dubia*, nitrogen fertilizers, harvesting time, solid biofuel, pellets, energy parameters, properties, environmental impact

## Abstract

Field studies with the large-stemmed plant *Artemisia dubia* (*A. dubia*) have been carried out at the Vėžaičiai Branch of LAMMC since 2018. According to three years of experimental results, annual dry matter (DM) yield varied from 7.94 to 10.14 t ha^−1^. Growing conditions, nitrogen application level, and harvesting time had statistically significant impacts on *A. dubia* productivity. The most important tasks of this article were to investigate and determine the factors influencing *A. dubia* plant biomass productivity and the evaluation of technological, power, and environmental parameters of plant biomass utilization for energy conversion and the production of high-quality solid biofuel pellets. For the experiments, six variants of *A. dubia* samples were used, which were grown in 2021. Plants were cut three times and two fertilization options were used: (1) no fertilization and (2) fertilization with 180 kg ha^−1^ of nitrogen fertilizer. These harvested plants were chopped, milled, and pressed into pellets. The physical–mechanical characteristics (moisture content, density, and strength) of the *A. dubia* pellets were investigated. During this study, it was found that the density in the dry mass (DM) of the pellets ranged from 1119.86 to 1192.44 kg m^−3^. The pellet moisture content ranged from 8.80 to 10.49%. After testing pellet strength, it was found that the pellets which were made from plant biomass PK-1-1 (first harvest without N fertilization) were the most resistant to compression, and they withstood 560.36 N of pressure. The dry fuel lower heating value (LHV) of the pellets was sufficiently high and was very close to that of the pine sawdust pellets; it varied from 17.46 ± 0.25 MJ kg^−1^ to 18.14 ± 0.28 MJ kg^−1^. The ash content of the burned pellets ranged from 3.62 ± 0.02% to 6.47 ± 0.09%. Emissions of harmful pollutants—CO_2_, CO, NO_x_, and unburnt hydrocarbons (C_x_H_y_)—did not exceed the maximum permissible levels. Summarizing the results for the investigated properties of the combustion and emissions of the *A. dubia* pellets, it can be concluded that this biofuel can be used for the production of pressed biofuel, and it is characterized by sufficiently high quality, efficient combustion, and permissible emissions to the environment.

## 1. Introduction

In the European Union, the increase in the amount of energy obtained from biomass processing is promoted [1]. An increase in the number of heat production facilities is expected, where biomass will be used for heat or electricity or combined production. Traditional wood biomass is the most commonly supplied, but new feedstocks are being introduced to the market in these sectors to meet anticipated future demand. A promising alternative to cover the limited availability of high-quality wood biomass is herbaceous biomass, which also reduces the cost of the raw material supply. In this context, the increasing importance of the raw material of herbaceous biomass from energy plants and agricultural residues, whose processing can be undertaken with sustainable conditions in the market of biomass raw materials, is expected [2]. The use of fossil fuels as the main source of energy is directly related to global climate change due to CO_2_ emissions, so it is necessary to look for new, cheap, and easily accessible sources of energy.

In order to understand the suitability of raw material biomass for energy production, it is important to determine the elemental composition of the raw material. Therefore, it is important to determine the amount of chemical elements in the plant biomass. Carbon, nitrogen, hydrogen, and oxygen are the most important components of solid fuel, and during the fuel burning process, oxygen and carbon react in an exothermic reaction forming the following compounds: CO_2_ and H_2_O [3].

Special attention should be paid to decentralized low-cost renewable fuels, which can be used primarily in those households that do not have access to gas or heat supplied from other sources. Better and more efficient use of biomass energy is considered a favorable option for reducing carbon emissions. Biomass pellet fuel is one of the most common and important ways of using biomass energy, so granulation appears to be a great opportunity to increase the competitiveness of biomass pellet fuel production in the future [4]. Various agricultural plant wastes can also be used for the production of solid biofuel [5].

It has been established by scientific analysis that the densification of herbaceous biomass with an addition of woody biomass (spruce sawdust) improves the combustion characteristics of the densified herbaceous biomass (reed canary grass), providing a faster thermal decomposition of biomass pellets with the increase of the average mass loss. When producing the pellets, the addition of woody biomass ensures a more complete combustion of volatiles and decreases the average mass fraction of polluting emissions (CO, NO_x_) in the products. A densified biomass mixture of reed canary grass and spruce sawdust (equal parts) has a lower ash content, a higher heating value, and an increased heat production rate and total amount of energy in comparison with herbaceous biomass [6].

In comparing the woody pellets, straw pellets, and herbaceous biomass pellets (switchgrass and miscanthus) from the combustion technique perspective, the burning of herbaceous biomass pellets can cause problems like corrosion, slagging, and fouling. When considering the scenario of the increase in energy consumption, the market for herbaceous biomass pellets is bright [7]. Polish scientists have investigated the use of crude plant biomass such as wormwood (*Artemisia ab-sinthium* L.), Canadian goldenrod (*Solidago canadensis* L.), and common sedge (*Tanacetum vulgare* L.) as biofuel sources. The mentioned plants are considered weeds and have many advantages, allowing for wider use for energy purposes, especially in cases where large areas of land are covered with weeds.

Clean, fully biogenic, renewable fuel is an interesting alternative to the usually expensive processed wood biomass, such as pellets or briquettes. The research results revealed that the studied species can be considered excellent sources of primary energy due to their high calorific value (over 16 MJ kg^−1^), low moisture content, low costs, and availability. The studied plants were particularly promising because they occupy a large part of uncultivated land and can be used for fuel in many homes without changing boilers and heating systems [8]. According to other Polish scientists’ works, an even higher heating value of 17.40 MJ kg^–1^ was determined for willow *Salix viminalis* pellets. The ash content was higher in pellets produced from the green biomass of the willow leaf sunflower, with 9.92% DM (dry material), and giant miscanthus, with 6.85% DM [9]. According to Lithuanian scientists, a perennial herbaceous energy plant, *Artemisia dubia Wall,* is a promising plant for the enhancement of renewable resources. The highest *A. dubia* productivity in the first year was as low as 4.92 t ha^−1^; in the second and third years, the biomass productivity of unfertilized swards increased to 23.12 and 14.86 t ha^−1^ of DM, respectively. The average moisture content was from 59.2 ± 0.83% to 10.98 ± 0.60% in the *A. dubia Wall* harvest. It is notable that nitrogen fertilization did not have a significant influence on crop productivity. The studies showed that the calorific value of *A. dubia* plant raw material was 18.53 MJ kg^−1^ on average. Ash content varied from 2.54 to 3.44% on average [10]. According to the results of another work, the calorific value of energy crops varies from 17.92 MJ kg^−1^ in the *A. dubia* case to 18.50 MJ kg^−1^ in the *C. sativa* case [11]. 

According to other studies in Lithuania, the herbaceous plants *M. giganteus* and *A. dubia* are potential energy crops in the northern part of temperate climate zones. The highest productivity of evaluated energy crops was achieved in *M. giganteus* (21.54 t ha^−1^) and *A. dubia* (17.86 t ha^−1^) swards. *S. hermaphrodita* (12.30 t ha^−1^) and the traditional crop *F. arundinacea* (10.99 t ha^−1^) produced the lowest biomass DM yield. The nontraditional energy crops *Miscanthus giganteus* and *A. dubia* presented a high concentration of carbon, cellulose, and lignin and a lower concentration of the inhibiting elements potassium and phosphorus compared to other evaluated crops. The biomass quality of these crops was consistent with the results obtained by other researchers and can be compared with woody short-rotation forests. A higher energy value was determined for *A. dubia* and *M. giganteus*. Evaluation of energy potential and energy efficiency showed differences between crops and was effectively proportional to their biomass DM yield but not to their energy value [12]. The growth of herbaceous plants in a monoculture for biomass production in a 3-year period had a positive balance for all types of organic fertilizations at both cutting frequencies [13].

The tested herbaceous feedstocks of triticale, cynara, and Jerusalem artichoke stalks or the production of solid biofuels exhibited comparatively poorer fuel properties than their woody counterparts in terms of carbon and lignin content, heating value, and ash contents, exhibiting significantly higher levels of Cl and alkali metals. Despite the less demanding requirements of non-woody pellets, only triticale complied with the quality standards for this type of solid biofuel [14]. In other research works, various species were compared, for example, stone lichen (*Cetraria islandica*), mistletoe (*Viscum album*), knotweed (*Polygonum aviculare*), wheatgrass (*Agropyron repens*), and knapweed (*Centaurium erythraea*). Results showed that the species are good resources as biofuel in the form of pellets. Of all species, the *Viscum album* presents the highest heating value and carbon content and the lowest moisture content, thus being the most suitable material for biofuel pellet manufacturing [15]. 

According to other studies, herbaceous biomass ensures easy flow characteristics and a stable milled biomass pelletizing process, while woody biomass with coarse and hard particles causes the pelletizing unit motor to be overloaded. For comparison, the highest specific energy consumption for pelletizing in woody residues was 206 kWh t^−1^, and herbaceous residues demanded 71 kWh t^−1^, but the significant negative features of herbaceous pellets were low bulk density (616 kg m^−3^), high moisture content (10.64%), and low net energy density (9.7 GJ m^−3^). In contrast, woody pellets have bulk density (663–722 kg m^−3^), lower moisture content (3–4%), and high net-energy density (12.3–13.2 GJ m^−3^) [16].

The findings from Forbes et al. [17] show that biomass fuel experiments demonstrate that the diverse nature of biomass fuels can be found even in fuels from the same genus and origin that have undergone the same processing methods. The predictive analyses for energy content were shown to correlate well with combustion results, confirming the applicability of those to raw fuel as indicators of combustion outcomes. Fuel N content was seen to influence NO_x_ emissions, most especially in the willow and to a lesser extent in miscanthus. 

Herbaceous plant pellets have high potential as an alternative biomass plant for energy purposes. It should be noted when discussing emissions from burning herbaceous plant pellets that those made from the perennial herbaceous mallow plant *Sida hermaphrodita* (L.) Rusby (‘Sida’) showed low CO emissions of 40 mg Nm^−3^, good burnout, and low slagging behavior; however, increased levels of NO_x_ and SO_2_ were also noted. Combustion displayed high CO emissions (1300 mg Nm^−3^), while SO_2_ values were below 100 mg Nm^−3^. The contents of HCl in the flue gas ranged between 32 and 52 mg Nm^−3^. High contents of alkaline earth metals, such as CaO, resulted in high ash melting temperatures of up to 1450 °C [18].

In this work, the main focus is directed to the assessment of new plant species and their potential for energy use. One of these plants, *A. dubia,* was studied, which, compared to common wormwood, grows significantly more biomass and provides the opportunity to produce a larger amount of biofuel. Overall, *A. dubia,* as a species, was studied as an allelopathic plant [19], including the study of its biomass feedstock for the food industry, phytochemistry, or pharmacology [20]. 

In Lithuania, relatively recently, *A. dubia* has been studied as an energy crop. Comparative studies have shown that the productivity of *A. dubia* is significantly higher than that of *A. vulgaris* [21]. The influence of mineral and organic fertilizers on the productivity of *A. dubia* was investigated. It should be noted here that the data of the conducted studies are quite different. The productivity of *A. dubia* varied greatly and depended on the soil characteristics of the study area [22,23]. However, we did not find any data on whether *A. dubia* was grown and studied as an energy crop in other parts of the world.

To date, experiments with *A. dubia* have also begun in Western Lithuania (LAMMC Vėžaičiai Branch). Since there are different soil types in Western Lithuania, naturally acidic soils prevail here. In this respect, there are many sites that are not suitable for traditional agricultural crops here. Viewed from the other side, to not compete with traditional food crops, different alternative non-food crops or energy crops might be grown on the land which is less suitable or unprofitable as traditional farming land [24]. For that reason, plantations for a sufficient new sort of *A. dubia* plant which grows well in these lands and without the use of fertilizers were established. In addition, these plants can produce a high enough yield of stem biomass that can be harvested using the same machinery used to harvest corn stalks, and this biomass can be used for energy purposes. 

The possibilities of *A. dubia* plant biomass utilization for energy conversion and the determination of energetic and environmental parameters have not yet been well investigated. So, the most important tasks of this article were to investigate and determine the factors influencing *A. dubia* plant biomass productivity and the evaluation of technological, power, and environmental parameters of plant biomass utilization for energy conversion and the production of high-quality, pressed, solid biofuel. Parameters that can influence biomass productivity and utilization are climatic conditions, soil quality, cultivation practices, harvesting techniques, technological parameters, environmental parameters, chemical composition, economic considerations, policy and regulatory frameworks, and a lot of others. A comprehensive study integrating the main factors can provide valuable insights into optimizing *Artemisia dubia* plant biomass uses. The main focus of this work is on assessing the technological and environmental parameters of *A. dubia* plant biomass use for pressed biofuel production.

## 2. Results and Discussion 

### 2.1. A. dubia Productivity and Characteristics of Samples

**Figure 1:**
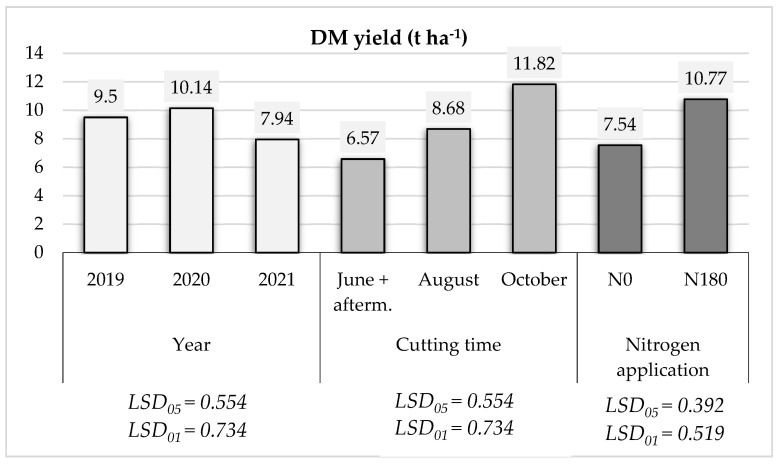
The average means of *A. dubia* productivity (t ha^−1^) and its dependence on year, harvesting time (the end of June, the middle of August, and the beginning of October), and nitrogen fertilization rate (N0 and N180).

The results presented in Figure 1 indicate that *A. dubia* DM yield during the successive three growing years highly depended on the growing year (i.e., meteorological conditions during vegetation), time of cutting, and nitrogen fertilization rate. The results indicate that *A. dubia* is a vigorously growing species. Planted once in 2018 and despite a period of moisture shortage in the topsoil layer in June, the DM yield in 2019 reached 9.52 t ha^−1^, on average. Accordingly, water and temperature regimes during the 2020 growing season were also favorable for plant growth and biomass accumulation, especially during the period of intensive biomass accumulation from May to July. In this respect, *A. dubia* productivity reached 10.14 t ha^−1^. In 2021, the weather for the first half of the vegetation cycle could be described as very warm with a relatively low amount of precipitation, especially in the months of June and July. Also, plants experienced a shortage of moisture in the upper soil layer. Here, the average DM yield reached 7.94 t ha^−1^ and was the lowest in comparison with the previous two seasons. 

The cutting of stems was performed as follows. Biomass was harvested three times: at the end of June (also including the aftermath performed in the beginning of October), in the middle of August, and in the beginning of October. In the fields where cuttings were performed two times per vegetation cycle, the average DM yield reached 6.57 t ha^−1^. It might be explained by the fact that once cut in June, *A. dubia* plants showed weak regrowth of their biomass (or aftermath) until the end of the vegetation cycle. Usually, the aftermath harvest was approx. 1.0–1.50 t ha^−1^ DM. Once harvested in the middle of August, DM productivity increased by 2.11 t ha^−1^, on average. Contrarily, when the plants had the opportunity to grow until October (or until the end of the growing season), their biomass increment was substantially higher—11.82 t ha^−1^ (or by 3.23 t ha^−1^ DM if compared to June + aftermath harvesting).

When growing in control treatments (N0), i.e., without nitrogen application, the average *A. dubia* biomass productivity per three-year period was 7.54 t ha^−1^ DM. Even without nitrogen fertilization, there were sufficient nitrogen reserves in the support plants’ physiological requirements during the first vegetation cycle. Further, in comparison with the plants growing without nitrogen fertilization (control treatment or N0), the use of the highest 180 kg ha^−1^ nitrogen rate (N180) (the fertilization was performed two times in split doses) caused an increase in DM yield up to 10.77 t ha^−1^ (or by 23.02%). Here, each 1 kg of nitrogen fertilizers caused an increase in productivity of 17.94 kg. 

The data presented in Figure 1 indicate that the data reliability corresponds to 95% and 99% probability levels.

### 2.2. Determination of Produced Mill Properties

**Figure 2:**
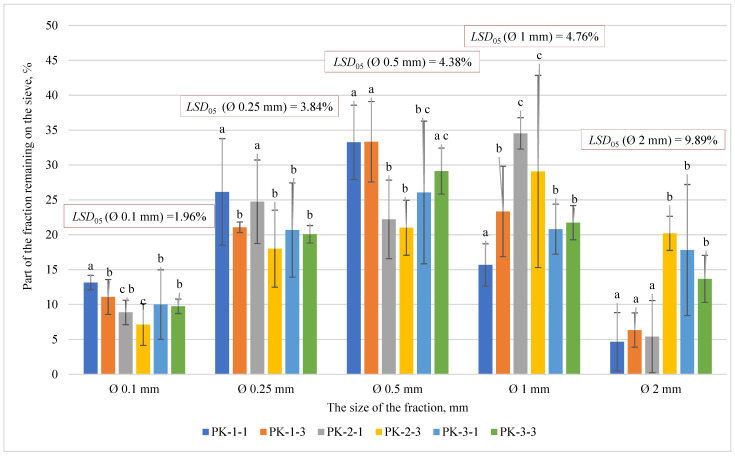
Results of *A. dubia* plant flour fractional composition. In the figure, any two samples with a common letter are not significantly different, as assessed using the least significant difference.

Before preparing the biomass of *A. dubia* stems for granulation, in the first step, the plant stems were chopped with a drum chopper into 15–20 mm particles. This chopped fraction was subsequently milled in a Retsch SM 200 (Retsch, Germany) hammer mill using a sieve with round holes of 2 mm diameter. Previous studies of biometric properties of fibrous plant granules and other herbaceous plant granules have shown that the produced pellets of well-milled fractions of flour with a fineness of 2 mm were of high quality and sufficiently strong [25]. The fractional composition of the produced flour is shown in Figure 2.

The matching letters shown in Figure 2 indicate no significant difference between different pellet types. Error bars represent the 95% confidence interval of the mean. A *t*-test was used for statistical analysis.

Having evaluated the fraction composition of the milled raw material, we may see that for the highest fraction of mass accumulated on a sieve with holes up to 1 mm in diameter (34.53 ± 2.24%) in the PK-2-1 series case, there was significant difference between all variants, except between PK-2-1 and PK-2-3 variants and also between PK-3-1 and PK-3-3 variants. A big amount of the fraction accumulated on the sieve with holes of 0.5 mm in diameter (from 33.33 ± 5.76% in PK-1-3 series case till 21.00 ± 3.94% in PK-2-3 series case). There was no significant difference between the PK-1-1 and PK-1-3 type raw materials or between the PK-2-1 and PK-2-3 variants, but significant differences were detected between the remaining types of raw materials. 

After plant milling, the moisture content of the flour was determined, which varied from 7.4 to 7.8%. The moisture content of the flour was too low for the production of biofuel pellets, so the flour was moistened to 13–15% before pressing the pellets.

### 2.3. Determination of Pressed Biofuel Pellet Properties

The properties of the pressed granules depend greatly on the granulation equipment used, the raw material and the quality of its preparation, the fractional composition of the flour, and the moisture content of the raw material.

#### 2.3.1. Pellet Biometric Properties

**Figure 3:**
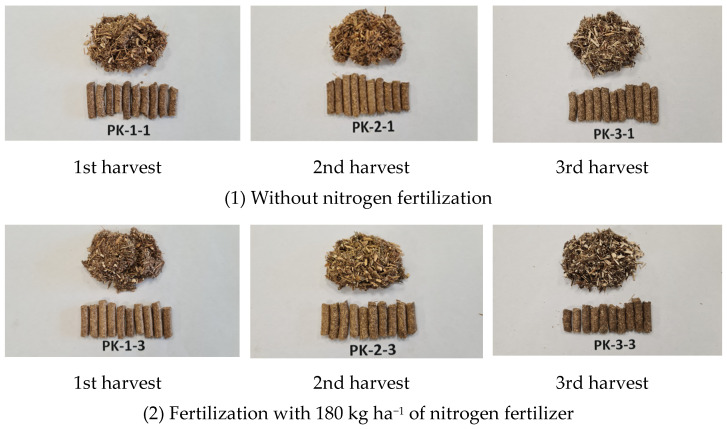
Biofuel pellets produced from *A. dubia*, which were fertilized with 0 and 180 kg ha^−1^ of nitrogen fertilizer and were harvested in different harvest periods.

The most important biometric properties of the produced and tested biofuel pellets using *A. dubia* chopped and milled plant stem biomass are presented in Figure 3.

**Figure 4:**
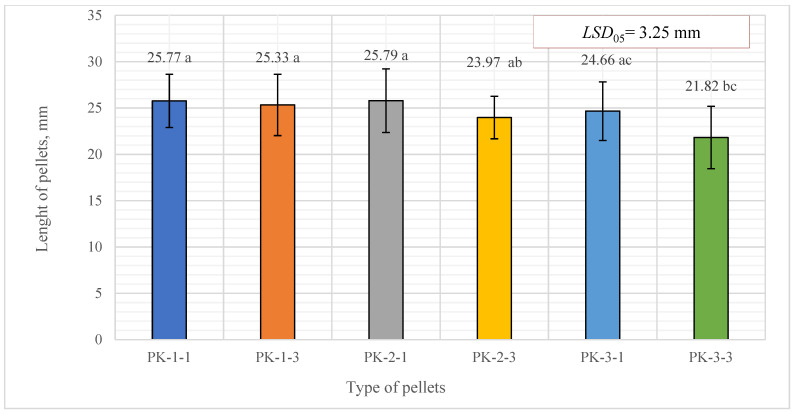
Length distribution for biofuel pellets produced from *A. dubia* plants. In the figure, any two samples with a common letter are not significantly different, as assessed using the least significant difference.

It was found that the average diameter of the biofuel pellet ranged from 6.22 to 6.39 mm and the average pellet length varied from 21.82 to 25.77 mm) (Figure 4). There was no significant difference between all types of pellets, except between the last three variants, PK-2-3, PK-3-1, and PK-3-3. In comparison, the studied pine sawdust pellets were longer, and their length reached 30.73 ± 0.88 mm.

The matching letters shown in Figure 4 indicate no significant difference between different pellet types. The error bars represent the 95% confidence interval of the mean. A *t*-test was used for statistical analysis.

#### 2.3.2. Pellet Moisture Content, Density, Ash Content, and Heating Value

**Figure 5:**
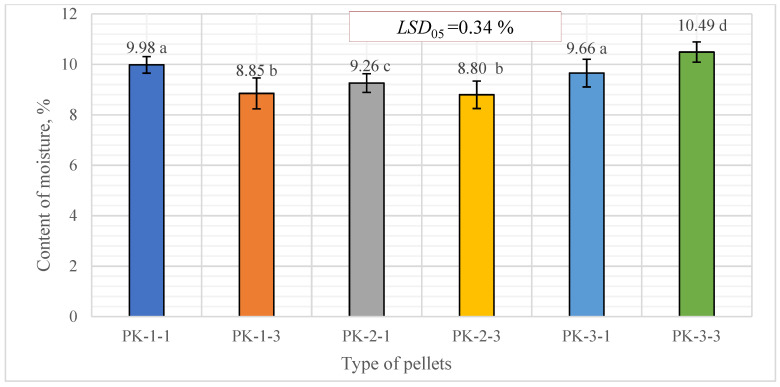
Distribution of moisture content for biofuel pellets produced from *A. dubia* plants. In the figure, any two samples with a common letter are not significantly different, as assessed using the least significant difference.

**Figure 6:**
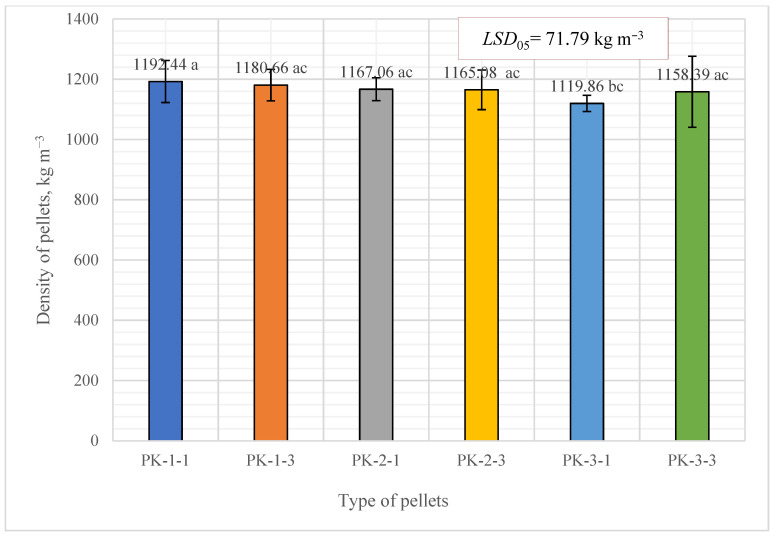
Average means for pellet density. In the figure, any two samples with a common letter are not significantly different, as assessed using the least significant difference.

**Figure 7:**
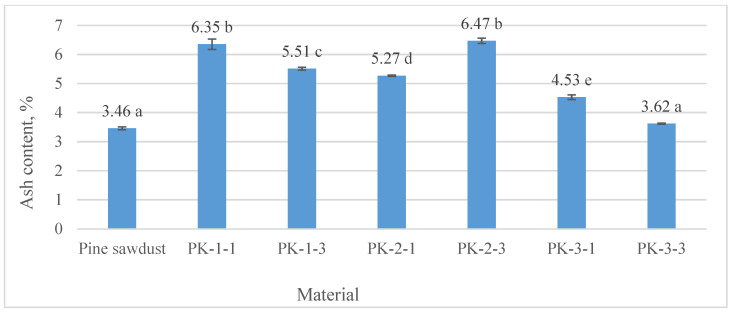
Determined means for ash content after *A. dubia* and pine sawdust pellet burning.

**Figure 8:**
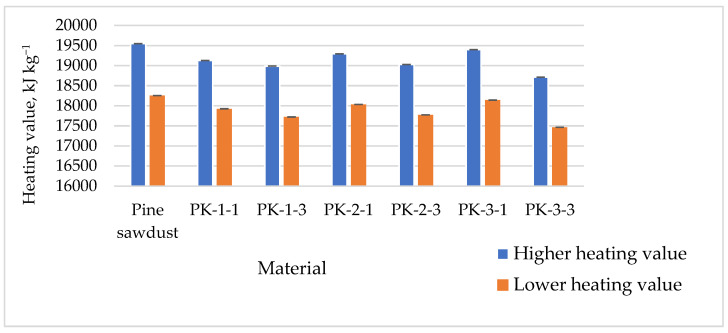
Means of *A. dubia* plant pellet heating values.

**Figure 9:**
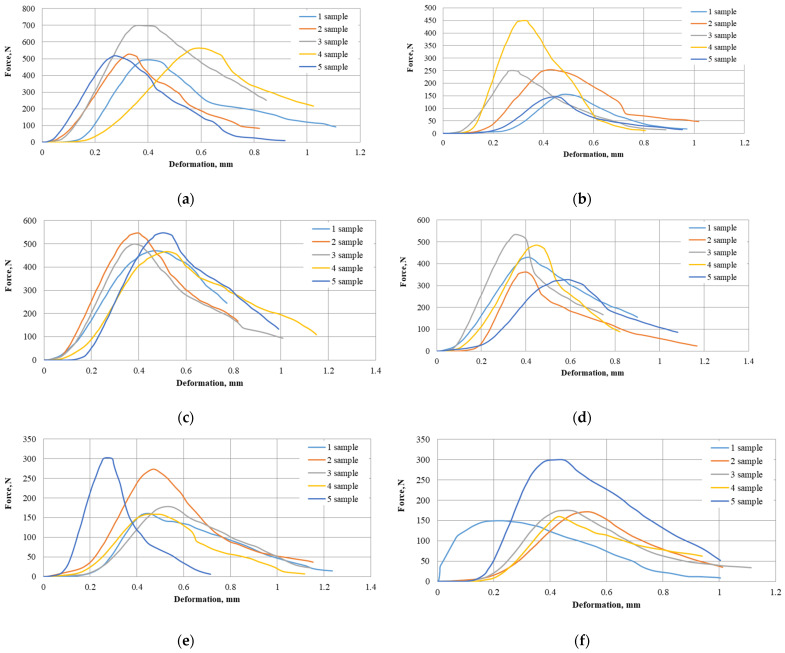
Curves produced for *A. dubia* pellet strength test: (**a**) PK-1-1; (**b**) PK-1-3; (**c**) PK-2-1; (**d**) PK-2-3; (**e**) PK-3-1; (**f**) PK-3-3.

**Figure 10:**
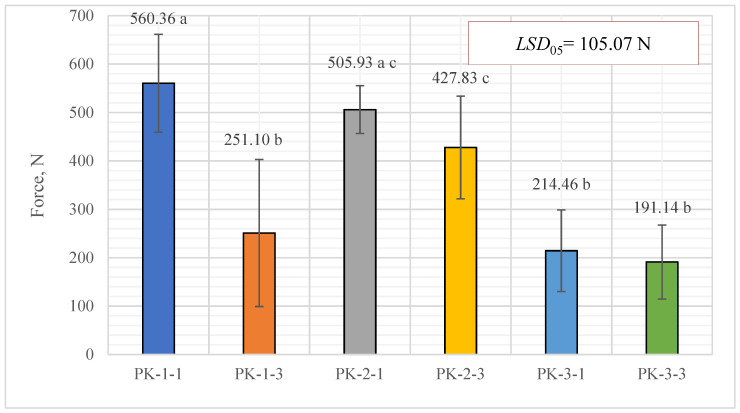
Comparison of the compressive strength of pellets produced from *A. dubia* plants. In the figure, any two samples with a common letter are not significantly different, as assessed using the least significant difference.

**Figure 11:**
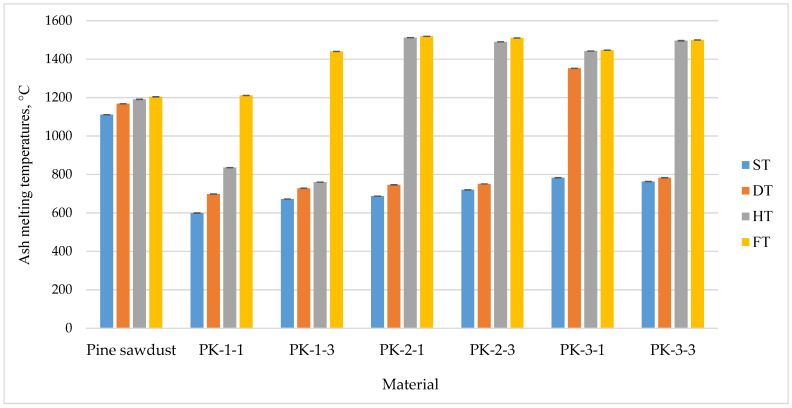
Variation in ash melting temperatures for *A. dubia* pellets.

**Figure 12:**
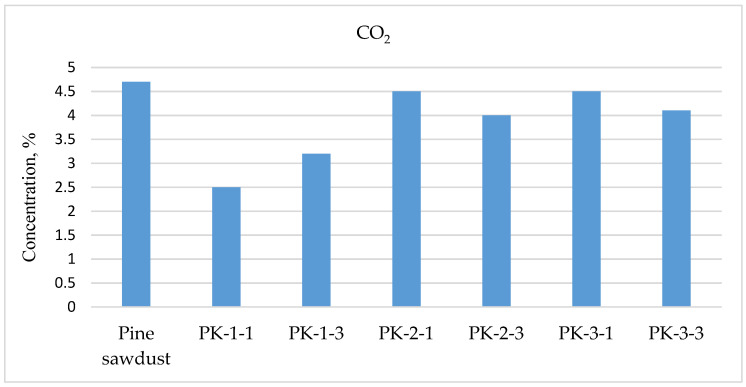
Determination of CO_2_ emissions from the burning of biofuel pellets.

The quality and properties of the granules are greatly influenced by their humidity. If the granules are too dry, they tend to absorb moisture from the environment and may swell and disintegrate, and such a mass of flour is difficult to burn in special pellet boilers. According to the studies conducted by Ungureanu et al. (2018), it is appropriate to recommend raw materials with a moisture content of 15–20% for the production of pellets [26]. Other researchers [27] studied pellets produced from a mixture of straw and willow and, based on the results of their research, indicated that the optimal moisture content of pellets for the combustion process is 6–8%. Therefore, it is obvious that it is important to determine the moisture content of biofuel pellets. Based on the results of the study of *A. dubia* and pine sawdust pellets, various important biofuel properties were determined. They are presented in Figure 5, Figure 6, Figure 7, Figure 8, Figure 9, Figure 10, Figure 11 and Figure 12.

After investigation of the most important properties of biofuel pellets produced from *A. dubia* biomass, it was found that the moisture content of all sorts of produced pellets was very similar and varied from 8.80 ± 0.55% to 10.49 ± 0.40%. The moisture content of pine sawdust pellets, which was tested for control, was lower and reached 7.50 ± 0.55% (Figure 5). There was no significant difference between the PK-1-1 and PK-3-1 variants or PK-1-3 and PK-2-3 variants, but a significant difference was found between the remaining variants. 

The matching letters shown in Figure 5 indicate no significant difference between the different pellet types. The error bars represent the 95% confidence interval of the mean. A *t*-test was used for statistical analysis.

The moisture content of the pine sawdust pellets, which was tested for control, was lower and reached 7.50 ± 0.55%.

The research results for the density of the pellets produced from *A. dubia* plants show that the highest density of biofuel pellets was produced from *A. dubia* plants (first harvest, type PK-1-1), and it reached 1192.44 ± 69.72 kg m^−3^. For comparison, the density of the pellets obtained from pine sawdust was 1100.90 ± 34.0 kg m^−3^. Summarizing the results for the density of the produced pellets, it can be stated that all types of biofuel pellets produced from *A. dubia* plants had a high density which exceeded 1000 kg m^−3^). 

The determined density results for the *A. dubia* plant pellets were statistically evaluated according to *LSD*_05_ tests and the significant difference of density was calculated. Significant differences between the PK-3-1 pellets and the other pellet types were detected. 

The matching letters shown in Figure 6 indicate no significant difference between different pellet types. The error bars represent the 95% confidence interval of the mean. A *t*-test was used for statistical analysis.

When evaluating the results of other researchers who investigated the density of biofuel pellets, it can be observed that sufficiently high density and strong pellets can also be produced from other large-stemmed herbaceous plants. For example, Maj et al. determined that the density of biofuel pellets produced from a mixture of corn cobs and corn husks reached 1150 kg m^−3^ and was very similar to our research results for *A. dubia* pellets [28]. Another researcher, Tulumuru et al., produced biofuel pellets from corn stover that reached a density of 1133 kg m^−3^ [29].

The ash content index of pellets is characterized by the amount of ash remaining after burning the pellets. If the amount of remaining ash is lower, then the quality and properties of the biofuel pellets are better [30]. 

After evaluating the ash content of the *A. dubia* pellets, it was found that it varied from 3.62 ± 0.02℅ (PK-3-3) to 6.47 ± 0.09 (PK-2-3). Very similar ash content was detected after burning of the type PK-3-3 and the control sample (pine sawdust pellets), which reached 3.46 ± 0.05% (Figure 7). There was no significant difference between the pine sawdust and PK-3-3 variants or between the PK-1-1 and PK-2-3 variants. It can also be noted that the lowest ash content of pellets obtained by burning was obtained from plants of the third harvest (without N fertilization—4.53 ± 0.08%; with fertilization of 180 N kg ha^−1^—3.62 ± 0.02℅). After evaluating the results of studies on the ash content of *A. dubia* pellets, it can be noted that they meet the main requirements of the standard [31]. 

Evaluating the results of other researchers’ studies on the ash content of nontraditional plant pellets shows that the amount of ash produced can be even higher. For example, burning palm kernel shells produced 10.67% ash, while rice straw pellets had an ash content of 12.03% [32]. 

The matching letters shown in Figure 7 indicate no significant difference between different pellet types. The error bars represent the 95% confidence interval of the mean. A *t*-test was used for statistical analysis.

Values for lower heating (LHVs) and higher heating (HHVs) for all types of pellets produced were very similar and sufficiently high, and these heating values varied from 17,920 to 18,250 kJ kg^−1^ (Figure 8). 

As can be seen, the heating values (kJ kg^−1^) for *A. dubia* biomass did not change statistically significantly with respect to cutting time. When evaluating the use of nitrogen fertilizers (N180), the calorific value of *A. dubia* was slightly lower. When evaluating the amount of nitrogen in the grown biomass, it was found that when nitrogen fertilizers were used, the amounts for Artemisia dubia biomass were higher than for the biomass grown in unfertilized soil [24].

When evaluating the results of research by other scientists using various plant biomasses, similar trends can be observed, indicating that the determined heating values can be similar. Ozturk et al. found that the higher heating value of corn pellets was quite high and could be as high as 18.11 MJ kg^−1^ [30].

Researchers from Croatia investigated the energy properties and biomass productivity of switchgrass (*Panicum virgatum* L.) in northwestern Croatia [33]. It was determined that, compared to autumn, switchgrass harvested in spring had higher quality biomass and its properties for biofuel preparation were improved: plant moisture decreased from 33.88% to 10.95% and ash content from 4.59% to 3.1%; calorific value also increased from 18.60 MJ kg^−1^ to 18.73 MJ kg^−1^ [33].

#### 2.3.3. Evaluation of *A. dubia* Pellet Strength and Resistance to Compression 

Biofuel pellet strength ensures that the fuel can be used and stored without breaking down into finer particles. The strength test curves for all the investigated series of pellets purposely show the character of the force variation in the different *A. dubia* raw material series types, as shown in Figure 9. After analyzing the deformation curves, it was observed that the maximum crushing force in the horizontal direction for sample No. 3 achieved more than 699 N when the deformation ranged from 0.35 mm to the point when the pellet completely disintegrated in the PK-1-1 series case, and the mentioned pellet series showed the greatest strength result compared to all other samples. 

The other samples in the PK-1-1 series case reached their maximum compressive forces in the zone of 494 N to 562 N, with deformation ranging from 0.27 to 0.57 mm. PK-2-1 deformed at a maximum compression force of more than 450 N in the sample No. 3 case, and the compression deformation was 0.33 mm. The remaining samples only reached 145 N to 250 N, with deformation ranging from 0.26 to 0.47 mm. The strength test results for the PK-2-1 series samples were evenly distributed from 466 to 547 N, with deformation ranging from 0.39 to 0.52 mm. In the PK-2-3 series, the samples ranged from 326 to 534 N, with deformation ranging from 0.40 to 0.59 mm. The PK-3-1 and PK-3-3 series pellets did not reach more than 301 N maximum compression force.

The experimental results for pellet compressive strength presented in Figure 10 show that the average strength of the PK-1-1 series pellets, with a semi-static stability of 560.36 ± 100.97 N in the horizontal direction, was found to be the most mechanically stable. In second place were the PK-2-1 series pellets, but there was no significant difference between the PK-2-1 and PK-2-3 series pellets. It can be said that both series of these granules are mechanically stable (505.93 ± 49.30 and 427.83 ± 106.15 N, respectively). The semi-static stability of the PK-1-3 pellets was 251.10 ± 151.97 N, PK-3-1 achieved 214.46 ± 84.26 N, and PK-3-3 achieved 191.14 ± 76.49 N, on average. There was no significant difference between all three mentioned variants. It should be emphasized that the difference compared with the most mechanically stable PK-1-1 series pellets was almost two-fold lower. The weaker pellets are still suitable for use as a biofuel, but there is a possibility that the pellets will break down faster during reloading and storage activity.

The matching letters shown in Figure 10 indicate no significant difference between the different pellet types. The error bars represent the 95% confidence interval of the mean. A t-test was used for statistical analysis.

#### 2.3.4. Pellet Elemental Composition and Ash Melting Temperatures

The elemental composition and ash melting temperatures determined for the *A. dubia* pellets are presented in Table 1. Research results for the determination of pellet elemental composition show that the biggest amount was detected for carbon C, which varied from 47.34 ± 0.12% for the first harvest with 180 kg ha^−1^ N fertilization (PK-1-3) to 49.36 ± 0.01% for the third harvest with 180 kg ha^−1^ N fertilization (PK-3-3). These values, the biggest values for carbon C, were very similar to those for pine sawdust pellets, which were 49.87 ± 0.11%.

The results for pellet ash melting temperatures show that ST and DT temperatures were lower than for pine sawdust pellets, except for the case PK-3-1 (1352 ± 0.63 °C). Other ash melting temperatures, such as HT and FT, were significantly higher than the temperatures for pine sawdust pellets, except for the cases PK-1-1 and PK-1-3, when ash melting temperatures reached only 836 ± 0.32 °C and 760 ± 0.37 °C. These values were similar to some sorts of straw, which are not recommended for use in biofuel production because use of this sort of biofuel can cause slag formation on the surfaces of the burning implements [34].

Visually, these ash melting temperature distributions and variations are very clearly visible in the presented Figure 11. 

After performing the correlation analysis, it became clear that the carbon and nitrogen amounts of the granules, as well as the amounts of oxygen, nitrogen, and hydrogen, were mostly inversely correlated (Table 2).

Reliable moderate to strong negative correlations were found between pellet carbon, oxygen, and ash content (Table 3). Hydrogen was more correlated with the ash melting temperature and ST, and sulfur correlated with the DT and HT. Nitrogen content was correlated with many investigated parameters: pellet density, ash content, ash melting temperature, ST, and DT. A reliable strong positive relationship was established between the moisture content of the granules and the amount of chlorine in them.

Correlation analysis of the data showed that the thermo-physical properties of the granules were poorly correlated with each other, except for pellet density, which correlated with ash melting temperatures ST and DT.

#### 2.3.5. Determination of Harmful Emissions from the Combustion of Produced Pellets

The results of studies of harmful emissions during the burning of biofuel pellets produced from *A. dubia* and pine sawdust pellets are presented in Table 4. 

These results show the sufficiently high quality of the produced biofuel and its suitability for use as a solid biofuel when trying to minimize environmental pollution and the negative impact on people.

According to the requirements of the standard [25], it was determined that the emissions of the produced *A. dubia* and pine sawdust pellets did not exceed the maximum permissible concentrations. However, it was observed that in sample PK-1-1, several indicators slightly exceeded the permissible limits: the detected concentration of CO gas was the highest and reached 8303 ppm, and the concentration of C_x_H_y_ gas was also too high and reached 1109 ppm.

Analyzing the results of the determined harmful emissions, it can be seen that the lowest concentrations of harmful gases were obtained when burning the control sample, pine sawdust pellets. Their values were obtained as follows: CO—188 ppm; NO_x_—111 ppm; and C_x_H_y_—9 ppm. However, when burning the produced control sample, the highest concentration of CO_2_ emissions was detected, reaching as much as 4.7% (Figure 12).

High concentrations of harmful gases were detected during the burning of early harvest sample PK-1-1, and only the CO_2_ emissions of this sample were determined to be the lowest and amounted to only 2.5%, which was almost two times lower compared to the control sample.

Analyzing the results of the determined harmful emissions of NO_x_, it can be seen that all investigated plant pellets did not exceed the maximum permissible levels. But the determined concentration of CO gas was too high for some plant species: the highest CO concentration was for plant species PK-1-1 and reached 8303 ppm, and concentrations that were too high were observed for plant species PK-2-3 (4121 ppm) and for PK-1-3 (3219 ppm), but these concentrations can be allowed for low-capacity boilers according to the requirements of other standards.

Emissions for other harmful gases, such as sulfur dioxide, were also investigated during the burning of *A. dubia* pellets, but in all samples, these emissions were very low or were not detected. When evaluating all the research results for the harmful emissions studies, it was observed that by delaying the harvesting time of *A. dubia*, lower emissions of harmful gases and, at the same time, a lower harmful impact on the environment were observed.

Summarizing these results, it is reasonable to recommend these plants, which grow well in the Lithuanian climate, to be grown and used for the production of pressed solid biofuel. However, in order to ensure that the burning of such biofuel has the least harmful impact on the environment, it is recommended to harvest Artemisia dubia plants for the production of granular biofuel as late in autumn as it is possible.

## 3. Materials and Methods

### 3.1. A. dubia Plant Growth in the Fields

Field trials were conducted with *Artemisia dubia* plants in 2019–2021. The plants were grown and investigated in Western Lithuania, on the eastern edge of the coastal plain (55°430′ N, 21°270′ E). The study field is a naturally acidic moraine *Bathygleyic Dystric Glossic Retisol* [35] with a clay content (<0.002 mm) of 15.0%. The determined average annual precipitation in the studied field was about 915 mm.

This field experiment was conducted according to the two-factor method. Factor 1 was harvest time and Factor 2 was nitrogen rates. *A. dubia* biomass was harvested 3 times: (1) at the end of June (PK-1-1 and PK-1-3); (2) in mid-August (PK-2-1 and PK-2-3); and (3) at the end of the growing season, that is, at the beginning of October (PK-3-1 and PK-3-3). The harvested area of all treatments was 3.3 m^2^.

Factor 2 was nitrogen rates. The seedlings were planted and fertilized at 0 and 180 N kg ha^−1^ rates. However, this article presented only two experiments, when nitrogen levels were 0 and when they were the highest amount, 180 kg ha^−1^. Each experiment was repeated 3 times.

### 3.2. Fractional Composition of Milled Biomass

To determine the suitability of *A. dubia* plants for biofuel production, at the beginning, the stems were chopped with a drum chopper into 5–7 mm long particles and then milled into flour with a Retsch hammer mill (Retsch, Germany). To assess the quality of the flour, its fractional composition was determined. For this purpose, 100 g of chopped and milled biomass samples of plant stems were used, which were sieved with a Haver EML Digital plus sieve shaker with a sieve set Retsch SM 200 (Retsch, Germany) by using sieves with the following mesh sizes: 0.25 mm, 0.5 mm, 0.63 mm, 1.0 mm, and 2.0 mm. The flour was sifted with a set of sieves for 1 min, the mass remaining in the sieves was weighed, and the percentage of every fraction was calculated. Each test was repeated 3 times.

### 3.3. Flour Pressing and Pellet Production

During this research, six variants of biofuel pellets were produced from *A. dubia* biomass, and softwood sawdust pellets were produced as a control sample. The research was carried out in the laboratories of Vytautas Magnus University Agriculture Academy. A 7.5 kW granulator “Peleciarka” (POLEXIM, Izdebki, Poland) with a horizontal matrix with 6 mm holes was used for production of granulated biofuel. After cooling for 15–20 min for pressed granules, their biometric, physical–mechanical, thermal, and other properties were investigated. All of these research studies were conducted using standard research methods and specialized equipment and devices [25,36].

### 3.4. The Biometric Indicators of the Produced Pellets

The biometric indicators of the pellets were determined by measuring their length and diameter in the central part of the pellets [37]. A digital Vernier caliper was used for this purpose (measurement accuracy 0.01 mm). Ten pellets from each type of produced pellet were selected for experimental studies. The mass of the pellets was estimated by weighing them on a KERN ABJ balance (weighing accuracy 0.001 g), and the average values of the measured mass of all tested pellet types with errors were calculated.

### 3.5. Pellet Moisture Content and Density

The moisture content of the pellets was determined using a standard methodology using a drying chamber [38]. The density of different types of granules was calculated based on the mass of the weighed granules and their calculated volume, knowing the length and diameter of the granules [25].

### 3.6. Tests on the Mechanical Strength (Compression Resistance) of the Pellets

Determination of the mechanical strength (compression resistance) of the pellets was performed using the Instron Universal Tester (INSTRON, USA) [39]. In this study, a cylindrical pellet was placed on a horizontal plane and acted upon vertically by a special rod, gradually increasing the load force. When the device was switched on, the pellet was subjected to a low (1 mm min^−1^) compression rate, and the loading force was stopped when the pellet was fully deformed or broken. With the help of the device’s computer program, the applied force and the displacement of the rod were recorded, and graphs illustrating the changes in the force acting on the pellet were drawn.

### 3.7. Pellet Thermal Properties, Heating Values, and Ash Content

These experiments were performed in the Thermal Equipment Research and Testing Laboratory of the Lithuanian Energy Institute (LEI). Based on standard methodologies, the lower and higher heating values (LHVs and HHVs) of the pellets, the values of chlorine and other elements, and the amount of ash after pellet burning were determined [24].

### 3.8. Characteristics of Ash Melting Temperatures

The characteristics of ash melting temperatures were determined by using a high-temperature Carbolite CAF digital furnace and by using the methodology presented in the Lithuanian standard [40]. This test is carried out by heating the ash sample in a special furnace, in which the sample changes its state and shape as the temperature of the sample is raised. According to the ASTM D 1857 standard, the changes in the shape of the prepared ash cone for the test, when it is burning in an oxidizing environment, are divided into the following four phases [41]

- Initial point of deformation (IT), when the sharp peak is rounded;
- Softening temperature (ST), when the ash cone deforms and its height decreases to the size of its diameter;
- Hemisphere temperature (HT), when the sample assumes a hemispherical shape;
- Melting point (FT), at which the ash melts and liquefies.

### 3.9. Emissions of Released Harmful Gases into the Environment

Harmful emissions into the environment were determined by burning pellets in a special low-power (5 kW) hearth-type stove, Astra P–5 (Astra, Lithuania). A sample mass of 5 kg of each type of produced pellets was used for burning. During pellet burning, the average exhaust gas temperature was determined, which varied between 110 and 200 °C. The produced harmful gases were measured by using combustion product analyzers Datatest 400 CEM and VE7. Each sample was burned for about 10–12 min, and the emissions of harmful gases into the environment—CO, CO_2_, NO_x_, and C_x_H_y_—were measured. Limit values for harmful emissions of biofuel burning facilities are determined by using the standards valid in Lithuania [42].

When evaluating and comparing the results of the conducted experimental studies, the properties of various types of granular biofuel were analyzed and evaluated, and the repeated values of the research data were statistically evaluated by one-way analysis of variance, correlation, and regression [43].

## 4. Conclusions

Six variants of *A. dubia* pellets and the control sample pine sawdust pellets for biofuel use were produced and tested. A field experiment was conducted according to the two-factor method. Factor 1 was harvest time. *A. dubia* was harvested three times: at the end of June (PK-1-1, 0 N and PK-1-3, 180 N); in mid-August (PK-2-1, 0 N and PK-2-3, 180 N); and at the beginning of October (PK-3-1, 0 N and PK-3-3, 180 N). Factor 2 was nitrogen rates. The seedlings were planted and fertilized by two nitrogen levels (0 and 180 N kg ha^−1^).

After the evaluation of pellet biometric and physical-mechanical properties, it was determined that the density in dry mass (DM) of the tested *A. dubia* pellets ranged from 1119.86 kg m^−3^ to 1192.44 kg m^−3^. The pellets which were made from *A. dubia* plant biomass PK-1-1 were the most resistant to compression; they withstood 560.36 N of pressure force.

The dry fuel lower heating values (LHVs) of the investigated *A. dubia* pellets varied from 17.46 MJ kg^−1^ to 18.14 MJ kg^−1^ and it was sufficiently high and very close to the LHV of the pine sawdust pellets. The ash content of the burned pellets ranged from 3.62℅ (PK-3-3) to 6.47℅ (PK-2-3).

The determined pellet elemental composition shows that the biggest amount was detected for carbon C, which varied from 47.34% to 49.36%. The biggest C values were very similar to that of the pine sawdust pellets, which reached 49.87%.

Analyzing the results for harmful emissions, it can be seen that they did not exceed the maximum permissible levels. The lowest concentrations of harmful gases were obtained when burning pine sawdust pellets: CO—188 ppm; NO_x_—111 ppm; C_x_H_y_—9 ppm.

Summarizing the results for the investigated *A. dubia* pellet properties of combustion and emissions, it can be concluded that *A. dubia* can be used for the production of pressed biofuel because it is characterized by high quality, efficient combustion, and permissible emissions.

## Figures and Tables

**Table 1:** Elemental characteristics of investigated *A. dubia* and pine sawdust pellets and ash melting temperatures.

Parameter	PK-1-1	PK-1-3	PK-2-1	PK-2-3	PK-3-1	PK-3-3	Pine Sawdust (PS)
*A. dubia* pellet elemental composition, %							
C, %	48.67 ± 0.20	47.34 ± 0.12	48.33 ± 0.09	47.52 ± 0.10	48.73 ± 0.11	49.36 ± 0.01	49.87 ± 0.11
N, %	1.22 ± 0.09	1.41 ± 0.13	1.00 ± 0.03	1.51 ± 0.01	0.68 ± 0.02	0.90 ± 0.01	0.47 ± 0.01
H, %	5.51 ± 0.01	5.79 ± 0.06	5.76 ± 0.09	5.75 ± 0.02	5.76 ± 0.02	5.73 ± 0.05	5.94 ± 0.03
S, %	0.07 ± 4.91	0.07 ± 8.77	0.06 ± 2.78	0.06 ± 11.49	0.05 ± 2.34	0.06 ± 5.49	0.06 ± 2.28
O, %	37.8	39.6	39.4	38.4	39.9	40.0	40.1
Cl, %	0.37 ± 9.81	0.30 ± 5.86	0.20 ± 10.04	0.25 ± 8.77	0.37 ± 9.81	0.38 ± 8.77	0.07 ± 1.31
*A. dubia* pellet ash melting temperatures, °C							
ST, °C	599 ± 1.56	672 ± 0.42	687 ± 0.62	720 ± 0.79	783 ± 0.54	763 ± 0.93	1111 ± 0.38
DT, °C	698 ± 0.81	728 ± 0.78	746 ± 0.76	751 ± 0.19	1352 ± 0.63	783 ± 0.90	1168 ± 0.48
HT, °C	836 ± 0.32	760 ± 0.37	1512 ± 0.19	1490 ± 0.19	1442 ± 0.39	1496 ± 0.87	1191 ± 0.59
FT, °C	1211 ± 0.58	1440 ± 0.39	1519 ± 0.28	1510 ± 0.37	1447 ± 0.29	1500 ± 0.19	1204 ± 0.47

**Table 2:** Correlations (r) between pellet chemical properties.

Independent Variables, x	Dependent Variables, Y					
	C, %	O, %	H, %	N, %	S, %	Cl, %
C, %	1.000	0.433	n	−0.880 **	−0.334	n
O, %	-	1.000	0.775 *	−0.676	−0.498	n
H, %	-	-	1.000	−0.479	−0.398	−0.724
N, %	-	-	-	1.000	0.587	0.330
S, %	-	-	-	-	1.000	n

Notes: *—significantly different from the control at *p* ≤ 0.05; **—significantly different from the control at *p* ≤ 0.01.

**Table 3:** Correlations between chemical and thermo-physical pellet properties.

Independent Variable x	Dependent Variables, Y							
	Humidity, %	Density, kg m^−3^	Ash Content, %	LHV, MJ kg^−1^	ST, °C	DT, °C	HT, °C	FT, °C
C, %	n	−0.635	−0.792 *	0.393	0.667	0.499	n	−0.502
O, %	n	−0.682	−0.883 **	0.344	0.618	0.564	0.305	n
H, %	−0.739	−0.744	−0.621	n	0.807 *	0.496	n	n
N, %	n	0.848 *	0.853 *	n	−0.759 *	−0.784 *	−0.301	0.347
S, %	n	0.723	0.459	n	−0.400	−0.758 *	−0.809 *	−0.351
Cl,%	0.901 **	0.496	n	0.398	−0.711	n	n	n

Notes: *—significantly different from the control at *p* ≤ 0.05; **—significantly different from the control at *p* ≤ 0.01.

**Table 4:** Emissions from the burning of pressed *A. dubia* and pine sawdust pellets.

Plant Species	CO_2_	CO	NO_x_	C_x_H_y_
	%	ppm	ppm	ppm
PK-1-1	2.5	8303	157	1109
PK-1-3	3.2	3219	150	307
PK-2-1	4.5	2447	169	206
PK-2-3	4.0	4121	206	495
PK-3-1	4.5	702	111	40
PK-3-3	4.1	2555	141	287
Pine sawdust	4.7	188	111	9

Notes: permissible limit values of harmful emissions. Lithuanian standard LST EN 303-5:2012; CO ≤ 3000 for 3rd class boilers; NO_x_ ≤ 750 mg Nm^−3^.

## Data Availability

Data are contained within the article.
